# Characteristics and Outcomes of Mechanically Ventilated Pediatric Patients in A Tertiary Referral Hospital, Addis Ababa, Ethiopia: Cross Sectional Study

**DOI:** 10.4314/ejhs.v31i5.2

**Published:** 2021-09

**Authors:** Tigist Bacha, Netsanet Tsegaye, Wagari Tuli

**Affiliations:** 1 Division of pediatric intensive care unit, Department of Pediatrics and Child Health, St. Paul's Hospital Millennium Medical College, Addis Ababa, Ethiopia; 2 Department of Emergency and critical care, University of Gondar, Gondar, Ethiopia; 3 Department of Emergency Medicine, Addis Ababa University College of Health Sciences, Addis Ababa, Ethiopia

**Keywords:** Mechanical ventilation, Mortality, Pediatric, intensive care, PIM II, Ethiopia

## Abstract

**Background:**

A few studies are available from Africa on the use of mechanical ventilation (MV) in the pediatric intensive care unit (PICU). Knowledge of the outcome of patients on MV is critical for better use of resources. We aimed to assess the characteristics and outcomes of mechanically ventilated pediatric patients in Tikur Anbessa Specialized Referral Hospital, Addis Ababa, Ethiopia.

**Methods:**

A cross-sectional study was done from September 2016 to February 2018. Data were reviewed from the patients' medical records. SPSS version 21 software was used for data entry and analysis.

**Results:**

There were 536 patients admitted to PICU; out of these, 202 (41.2%) were on MV. Sixty-three-point six percent of the participants were males and 130 (59.1%) died. The most common indications for the initiation of MV were respiratory problems 46 (20.9%) and 30.59/1000 ventilator days developed complications. Ventilator-associated pneumonia accounted for 18.6% of the complications with 20.9/1000 ventilator days. Survival of medical cases was better than the surgical cases (including trauma); [AOR= 0.13, 95% CI (0.04–0.41)] and those who have MV for more than 3 days are 79% more likely to die (p=0.003). Those who have multi-organ dysfunction syndrome (MODS) [AOR= 0.181, 95% CI (0.08, 0.412)] and high PIM II severity score [AOR= 35, 95% CI (1.7,11)] had higher mortality rate.

**Conclusions:**

higher PIM II score, MODS, length of stay, and being a surgical patient increased the risk of mortality. Early resuscitation and thorough follow up of these ventilated patients are necessary.

## Introduction

Mechanical Ventilation (MV) is a life-supporting strategy used at the time of either impending or acute respiratory failure with the aims of improving gas exchange and decreasing work of breathing (1, 2). Given the high load of respiratory problems being the primary reason for admission to the intensive care unit in low resource countries (LRIC), there is a high need for proper use of MV (3–8). There is a disparity of available mechanical ventilators and trained health providers between the LRIC and high resource income countries (HRIC). Most ICUs have no adequate mechanical ventilators in LRIC (3). The usual compelling argument against the provision of ventilators in resource-poor settings is it's cost and the inadequacy of current systems to appropriately care for patients on ventilators and the ventilators themselves (3).

The percentage of children receiving MV in PICUs ranges from 17–64% in developed countries where pediatric intensive care medicine are a well-established discipline of medicine (2). There is a great scarcity of data from African countries regarding the use of MV in PICUs. The incidence of utilizing MV in children in Egypt was 32.8 % (9). The study in Nepal showed that out of the 16 pediatric ICUs, 32% had only one functioning mechanical ventilator and another 38% had two ventilators, the other units had 3–6 ventilators (10).

Despite its important role, MV is associated with poor outcomes and might lead to complications like shock, ventilator-associated pneumonia (VAP), pulmonary hemorrhage, pneumothorax, atelectasis, and also side effects of medications (e.g. sedatives and analgesia) (2,11). Many studies in LRIC have revealed that the mortality rate ranges from 40–60% in mechanically ventilated children. A study in the PICU of Aga Khan University Hospital in Pakistan found that the mortality rate among mechanically ventilated patients was 30.5% and the complication rate was 9.4% (1). A report from Nepal revealed a 34.1% mortality rate (10).

The scarce resources in Ethiopia made physicians choose very difficult rationing bedside decisions because of a lack of resources like intensive care beds and mechanical ventilators(12). The information on patient characteristics and outcomes in patients requiring MV is critical for better use of resources and clinical decisions for the limited pediatric intensive care unit (PICU) beds (3, 13). However, this information regarding PICU MV is not well dealt within our setting. Therefore, the present study aimed to assess the characteristics and outcomes of mechanically ventilated pediatric patients in Tikur Anbessa specialized referral hospital, Addis Ababa, Ethiopia.

## Methods and Materials

**Setting and study period**: The study was conducted from September 2016 to February 2018 at Tikur Anbessa Specialized Hospital which is the largest referral and teaching hospital in Addis Ababa, the capital city of Ethiopia. The hospital used to manage children either in adult medical or surgical ICU. Starting from 2012 the first two pediatric emergency and critical care physicians for a country established a separate four-bed ICU which is the first PICU in Ethiopia. These two physicians cover both emergency and intensive care units during the day and on-call during the night while the nights are covered by pediatric residents. Nurse to patient's ratio was 1:2. There is no respiratory therapist.

Each PICU bed has mechanical ventilation and is equipped with a monitor along with end-tidal CO2 monitoring. This unit shares a portable X-ray machine with the adult ICU which is in the next door. Philip V200 mechanical ventilator was used for respiratory support. Mechanical ventilation in all patients were initiated through an endotracheal tube. The modes of MV mostly used were synchronized intermittent mandatory ventilation (SIMV) either volume or pressure limited with pressure support (PS), assist control pressure-controlled ventilation AC/PCV, assist control volume-controlled ventilation AC/VCV. The other parameters set depending on the patient's condition as the reaction of inspired oxygen (FIO2), Peak end-expiratory pressure (PEEP), Peak inspiratory pressure (PIP), and Tidal volume (TV). Monitoring the subjects on MV was done with clinical examination and since arterial blood gas was not available pulse oximeter and end tidal carbon dioxide monitor were used to monitor oxygenation and ventilation of patients respectively. A chest X-ray was ordered on demand.

All children on mechanical ventilation were sedated with intermittent doses of diazepam and morphine (which mostly available in PO form). Rarely, thiopental and propofol were used. Neuromuscular blocking agents had never been used. Sedation by continuous infusion was not used because of the lack of infuser pumps.

**Study design and population**: The institutional based cross-sectional study design was employed by using a review of the patient's medical charts. All pediatric patients who were mechanically ventilated in the pediatric ICU of Tikur Anbessa Specialized Referral Hospital (TASH) for at least 24 hours during the study period were included whereas those patients with incomplete charts and lost charts were excluded from the study.

**Data collection procedures**: The instrument used to collect the data for this study was a structured data abstraction tool developed from the literature review. The tool has three parts: socio-demographic characteristics of the participants, characteristics of mechanically ventilated patients, and patient outcome. Then PIMII calculator was used for calculating the PIM2 score (14).

**Statistical analysis**: Data were checked for completeness and coded manually and entered into SPSS version 21 for analysis. Both descriptive and analytical statistical procedures were utilized. Descriptive statistics like percentage, mean, median, and standard deviation were used for the presentation of characteristics of mechanically ventilated patients; and tables were used for data presentation. Binary and multivariable logistic regression models were used with 95% CI and p-value less than 0.05 taken as significant.

**Operational definition**: Ventilator-associated pneumonia was diagnosed in patients on MV for more than 48 hours with a new persistent infiltrate on chest radiograph and at least 3 of the following; fever, leucopenia or leukocytosis, increased sputum production, rales, cough or worsening gas exchange (8).

**Ethical consideration**: The ethical clearance was obtained from Addis Ababa University, College of health Sciences, departments of Emergency Medicine and Pediatrics and the child Health research and publication Committee. Additionally, the confidentiality of all the data was seriously respected by not mentioning patients' identifiers in the questioner and unauthorized individuals were not allowed to access the data which was collected by using a password-protected computer.

## Results

There were 537 patients admitted to the PICU in two and a half years and 220 patients met the inclusion criteria. Most of the study subjects (39.1 %) were younger than 1 year and 63.6% of the participants were males (Table 1).

**Table 1:** Clinical characteristics of the study population

Variable		Frequency	Percentage
Age	< 1	86	39.1
	1–5	74	33.6
	6–12	60	27.3
Sex	Male	140	63.6
	Female	80	36.4
Length of stay in days Mean +SD)= (9.33 +13.33)	1–3 days	93	42.3
	4–7days	63	28.6
	>8days	64	29.1
PIM 2 score Mean+ SD)=(20+23.9)	Range (1–91)		

**Clinical presentation**: The source for admission to PICU was from the pediatric emergency department (102; 46.4%), from the operation room (57; 25.9%), from inpatient units (47; 21.4%), and from another hospital (14; 6.4%). Regarding the general indication of PICU admission, 160 (72.7%) were medical patients and 60 (23.7%) were surgical patients. From 149 (67.7%) of the children who were screened for HIV and 4 (1.8%) were positive. The most common indication for the initiation of mechanical ventilation was respiratory problems 46 (20.9%) (Table 2). The mean weight in kilogram was 12.73±9.12 (mean±SD) and Glasgow Coma Scale (GCS) of patients at admission was less than 8 in 73 (33.2%).

**Table 2:** Etiology of admission of the study population

Variable (n, %)		Frequency	Percentage
	Status epilepticus	12	5
Neurology (41,18.7)	Brain abscess	6	2.7
	Traumatic brain injury	6	2.7
	Meningitis	7	3.2
	Stroke	4	1.8
	Brain tumor	3	1.4
	Intra cranial hemorrhage	2	0.9
	Spinal cord injury	1	0.5
	Pneumonia	15	6.8
Respiratory (46, 20.9)	Tuberculosis	8	3.6
	FBA	6	2.7
	Apnea	4	1.8
	Aspiration pneumonia	4	1.8
	Lung contusion	2	0.9
	PCP	2	0.9
	HAAD	1	0.5
	HAP	1	0.5
	PTE	1	0.5
	Pulmonary edema	1	0.5
	Severe croup	1	0.5
	Post cardiac arrest	14	6.4
Cardiac (29, 13.2%)	Congenital heart disease	10	4.6
	Congestive heart failure	3	1.4
	SVT	2	0.9
Sepsis (34, 15.5%)		34	15.5
Renal (10, 4.5%)	Acute renal failure	6	2.7
	Chronic renal failure	4	1.8
Neuromascular (19, 8.6%)	Guillain Barrie syndrome	19	8.6
	Superior mediastinal mass	5	2.3
Malignancy (8, 3.6%)	Anterior mediastinal mass	2	0.9
	Tumor lysis syndrome	1	0.5
Other (33, 15%)	Post-operation	28	12.7
	Poisoning	2	0.9
	Diabetic ketoacidosis	1	0.5
	Uremic encephalopathy	2	0.9

FBA: foreign body aspiration PTE: Pulmonary thromboembolism, HAP: Hospital-acquired pneumonia, HAAD: Hyperactive airway disease, PCP: Pneumocystis carinii pneumonia. SVT: Supraventricular tachycardia

**Mode of ventilation and weaning method**: The access to airways was through endotracheal tubes in most cases, while 6 cases were tracheostomized (2.7%). Regarding the modes used at the initiation of mechanical ventilation, SIMV + PS, SIMV/VSV, AC/PCV, AC/VCV, and BIPAP/CPAP were used in 80%, 8.6%, 4.5%, 3.2 %, and 3.6% of cases respectively. The duration of MV ranged from (1–90) days with a median of 4.4 with IQR (2–10.3). The weaning methods recorded for these patients were CPAP alone 54(24.5%), direct oxygen trial 14(6.4%), PS with CPAP 12(5.5%), and unplanned accidental extubation 3(1.4%).

**Complications**: Complication occurred in 60 (27.3%) patients that is 30.55 per 1000 ventilation days, categorized as VAP 41(18.6%) (20.9/ 1000 ventilation days), Pneumothorax 15 (6.8%) (7.6 /1000 ventilation days), atelectasis 11(5%) (5.6/1000 ventilation days) and post-extubation stridor 1(0.5%) (0.5/1000 ventilation days). More than one complication occurred in 8 (3.6%) patients. About half (57.3%) of the patients developed multiple organ dysfunction Syndrome (MODS).

**Outcome of patients**: Among the total study participants, 130(59.1%) died where sepsis 59 (26.8%) was the leading cause of death. From the survivors, 75(34.1%) transferred to the ward, 12(5.5%) left the hospital against medical advice and 3 (1.4%) were discharged (Table 3).

**Table 3:** Outcome of mechanically ventilated pediatric patients in TASH PICU, (n=220) 2018

Variable		Frequency	Percent
Outcome	Survived	90	39.9
	died	130	59.1
Cause of death	Sepsis	59	26.8
	ARDS	30	13.6
	Brain death	17	7.7
	Intractable heart failure	12	5.5
	Other**	13	5.9

** Renal failure, Surgical site bleeding, Tension pneumothorax, Blocked ETT, Intracranial hemorrhage

**Logistic regression results**: Medical cases survived better than the surgical cases (including trauma); [AOR= 0.13, 95% CI (0.04–0.41)] and those who have mechanical ventilation for more than 3 days were 79% more likely to die than those with less than 3 days of ventilation; (p=0.003). The patients without MODS were more likely to survive than those with MODS [AOR= 0.181, 95% CI (0.08, 0.41)]. The patients who had a high PIM II score had a higher death rate [AOR= 4.35, 95% CI (1.7, 11)] (Table 4).

**Table 4:** Logistic regression analysis of associated factors with mortality in mechanically ventilated pediatric patients in TASH pediatrics ICU (n=220) 2018

Variable	Categories	Final Outcome		COR (95 % CI)	AOR (95% CI)

		Survived (n=90)	Died (n=130)		
Age	< 1 year	31	55	1.27 (0.64, 2.49)	
	1–5 year	34	40	0.84 (0.42, 1.67)	
	6–12 year	25	35	1	
Sex	Female	32	48	0.94(0.54, 1.64)	
	Male	58	82	1	
Type of cases	Medical	48	112	5.44(2.933, 10.73) *	0.127(0.37,0.41) **
	Surgical	42	18	1	
Admission diagnosis	Neurology	18	23	0.61(0.248, 1.49)	
	Respiratory	18	28	0.61(0.26, 1.41)	
	Cardiac	11	18	0.45(0.18, 1.14)	
	Sepsis	6	28	0.20(0.07, 0.58) *	
	Neuromuscular	15	4	3.42(0.97, 11.96) *	
	Malignancy	2	6	0.30(0.055, 1.67)	
	Renal	2	10	0.20(0.07, 0.58) *	
	Other	23	10	1	
Indication for MV	Respiratory failure	3	15	25(2.10, 29.28) *	
	Cardiovascular	27	34	6.29(0.69, 7.14) *	7.149(1.30,39.48)
	Neurology	55	80	7.27(0.83, 63.97)	
	Other (post op)	5	1	1	
Length of stay on MV	1–3 days	26	67	0.2 (0.11, 0.43)	0.19(0.07,0.57) **
	4–7	23	40	0.32(0.16, 0.67)	0.26(0.09,0.77) **
	> 8	41	23	1	
Comorbidities	SAM	6	6	1.0(0.167, 5.98)	
	Malignancy	14	23	0.609(0.131, 2.83)	
	HIV	1	3	0.33(0.02, 4.74)	
	CHD	10	17	0.58(0.12, 2.81)	
	Renal	1	3	0.33(0.23, 4.7)	
	Other	4	4	1	
Modes MV	SIMV/VCV	7	12	2.9(0.59, 14.06) *	
	AC/PCV	2	8	4.3(0.51, 36.9) *	
	AC/VCV	1	6	0.24(0.2421, 0.05) *	
	BIPAP/CPAP	6	2	1	
CPR before admission	Yes	12	27	1	
	No	78	103	1.7(0.81,3.57)	
Complication of MV	Yes	33	26	0.44(0.24, 0.8) *	
	No	57	103	1	
MODS	Yes	29	97	6.18 (3.4,11.18) *	0.181 (0.08,0.41) **
	No	61	33	1	
PIM II Score	0–9	54	50	0.417 (0.24, 0.72) *	4.35(1.7, 11) **
	9–91	36	80	1	

Mean and SD) (20, 23.9), MIN/MAX (1–91)

* P value < 0.2 for COR

** P value <0.05 for AOR

## Discussion

There is a scarcity of mechanical ventilators globally and it is exaggerated in Africa where fewer working ventilators in public hospitals available which makes its appropriate use critical (3). There is limited available data from African countries regarding the use of MV in the pediatric intensive care units. Even if most data from resource-limited setting are underreported; there are a high burden and mortality of respiratory failure in LRIC compared to HRIC, the provision of mechanical ventilators help save lives if implemented in a thoughtful fashion (3).

There were 536 Patients admitted during the study period with 202 (41.2%) were supported by mechanical ventilation. In a previous study in Gondar University the Northern part of Ethiopia, 10% of pediatric ICU admissions required MV (15). On the other hand, children admitted to the general ICU of the university hospital southeastern part of Ethiopia (Jimma) was 37% (16). Both of these findings are lower than the current study. This may be due to Tikur Anbessa is the last referral hospital in Ethiopia where more complicated cases are transferred. Other studies reported a varying incidence of MV use in PICU: 30% in 16 United States PICUs (17); in Egypt of 32.8 % (9), 34.6% in an Italian study (18), 50.7% in Pakistan (1) and 52% in Sri Lanka (19).

This study identified that respiratory (20.9%) was the most common indication for admission. A retrospective follow- up study in Turkey and another multicenter study showed acute respiratory failure was a primary reason for MV 59.18%, and 72%, 64.8% respectively (4, 20,21). However, our finding differs from a study done in a prospective observational study in Cairo in which the main indication for MV was neurologic cases 38.9% (9) and the discrepancy might be due to respiratory diseases that are common in Ethiopia and is one of the top causes of mortality in the country for children younger than 5 years of age (22).

This study found that SIMV was the most commonly used MV mode (80.0%). Similarly, the retrospective review in Pakistan (1) and prospective descriptive study in India (2), Egypt (9), Turkey (21), and Bangladesh (23) reported the commonest ventilator mode to be SIMV. Several published reports also found that SIMV the most commonly used mode of MV in multiple PICUs (17). The weaning method employed was CPAP alone 24.5% whereas the study of the group from Cairo showed that pressure support (PS) with CPAP was the preferred method of weaning in 74.7% of the cases (9). The difference could be due to the preference of the physicians and patients' capacity of maintaining their saturation on both methods.

The length of MV support in this finding showed a median of 4.4 days [IQR 2–10.3] and a mean of 9.3 (±13.33) days. The duration of MV ranged from 4–9 days in other reports. The median duration of MV was 3.1, 4.5, 5, 9 days in London Ontario (24), in Italian (18), Latin America (6), and Cairo (9) respectively. This variation could be due to differences in hospitals differences in cases mix, health provider staffing, and technologies.

We identified 60(27.3%) and 30.59 /1000 ventilator days developed complications. It is lower than the reported in Cairo (39.9%) and also 40% in Principi et al (24). This study showed VAP of 18.6% with 20.9/ 1000 ventilator days similar to Meligy et al where VAP had accounted for 20.19 per 1000 ventilation days (9). Higher values VAP reported in (36.2%) India (2). The atelectasis occurred in 5% in this study which is similar to (4.6%) in Pakistan (1) and (4.4%) in Egypt (9).

Logistic regression analysis reflected, predictors of mortalities were the presence of MODS; higher severity score, surgical rather than medical cases (including trauma), and pronged duration of MV. Our MODS rate of 57.3% of the cases higher than studies done in (7.6%) India (2) and (41.3%) Egypt (9). The higher discrepancy in our study might be due to delayed admission to PICU, a limited early resuscitation practice in our setting which is a crucial method of preserving organs from failing.

Prolonged MV more than 3 days were 79 % more likely to die than those of less than 3 days ventilated; (p=0.003). This is similar to the Pakistan study where prolonged mechanical ventilation (>10days) is an important predictor of mortality (1). Similarly, those who are on MV died more in Italian study than those who are not MV (18). The longer duration of MV is associated with increased mortality. This might be pronged ventilation will expose the patient to MV complications like increased risk of ventilator associated pneumonia, pneumothorax, etc.

A higher severity scores showed higher mortality in our study and which is similar to multiple other studies. Surgical cases die more than medical cases because we included those severe traumatic injuries in this list. This study revealed that the mortality rate was 59.1% which is higher than in Czech Republic 3.5 %( 27), in Italy 6.7% (18), in sir Lanka 27.6% (19), in Pakistan 30.3% ( 1), India 43.8%( 2) and in Egypt study(9) respectively. Also, it is higher than Faris et al from study of 36 Picus of seven countries 15.6% (30), The report in developed countries ranged from 1.6 – 15% (4–7, 18,19, 28). Sepsis (26.8%) and ARDS (13.6%) were among the common causes of mortality in our study Dahlem et al also showed the same (29).

These higher rates of mortality with severe PIM score and MORD speak about late admission of PICU patients may be because of the limited PICU bed and ventilators, delayed recognition and resuscitation of critically ill children. Lack of PICU trained enough staff could be the reason for poor outcomes (30). Limited knowledge of health providers on the use and management of mechanical ventilator; unavailability of basic tests for mechanical ventilators like blood gas and other necessary drugs all these could have contributed to the poor outcome of ventilated children.

This study identified that the mortality rate of mechanically ventilated pediatric patients in Tikur Anbesa Specialized Hospital. The main reason for the initiation of MV at PICU was respiratory failure and predictor of mortality are higher PIM II score, pronged duration of MV, the presence of MODS; the surgical case with nonmedical score were significant predictors of PICU mortality.

Early recognition, resuscitation, and transfer of critically ill and injured patients could prevent and improve the outcome of ventilated MODS and surgical (including trauma) patients. Meticulous follow up and care of patients with higher PIMII score patient is important. This will be only possible if the number of available pediatric intensive care unit service increased in the country.

Secondary data were used for this study; so that it was difficult in getting all the necessary data that are important for the study like anthropometry measurements. None of the patients had a blood gas analysis.
